# Redox Specificity of 2-Hydroxyacid-Coupled NAD^+^/NADH Dehydrogenases: A Study Exploiting “Reactive” Arginine as a Reporter of Protein Electrostatics

**DOI:** 10.1371/journal.pone.0083505

**Published:** 2013-12-31

**Authors:** Pooja Gupta, Mohamad Aman Jairajpuri, Susheel Durani

**Affiliations:** 1 Department of Chemistry, Indian Institute of Biotechnology Bombay, Mumbai, India; 2 Department of Biosciences and Bioengineering, Indian Institute of Technology Bombay, Mumbai, India; 3 Department of Biosciences, Jamia Millia Islamia, New Delhi, India; Oak Ridge National Laboratory, United States of America

## Abstract

With “reactive” arginine as a kinetic reporter, 2-hydroxyacid dehydrogenases are assessed in basis of their specialization as NAD^+^-reducing or NADH-oxidizing enzymes. Specifically, M4 and H4 lactate dehydrogenases (LDHs) and cytoplasmic and mitochondrial malate dehydrogenases (MDHs) are compared to assess if their coenzyme specificity may involve electrostatics of cationic or neutral nicotinamide structure as the basis. The enzymes from diverse eukaryote and prokaryote sources thus are assessed in “reactivity” of functionally-critical arginine as a function of salt concentration and pH. Electrostatic calculations were performed on “reactive” arginines and found good correspondence with experiment. The reductive and oxidative LDHs and MDHs are assessed in their count over ionizable residues and in placement details of the residues in their structures as proteins. The variants found to be high or low in ΔpKa of “reactive” arginine are found to be also strong or weak cations that preferentially oxidize NADH (neutral nicotinamide structure) or reduce NAD^+^ (cationic nicotinamide structure). The ionized groups of protein structure may thus be important to redox specificity of the enzyme on basis of electrostatic preference for the oxidized (cationic nicotinamide) or reduced (neutral nicotinamide) coenzyme. Detailed comparisons of isozymes establish that the residues contributing in their redox specificity are scrambled in structure of the reductive enzyme.

## Introduction

Advances in genomics place a premium on the methods to assess native proteins in physical properties of their functionally critical centers. This is important in characterizing the basis of protein-structure adaptation for specific functional roles. A method ideal for the study involves the prototropic groups assessed in their ionization equilibria in the native protein. Assessing effects of structure mutation, ionic strength, and pH variation illuminates the structure to function relation [1], [2]. This powerful method however has limits since only few of the ionizable residues of protein structure, notably His, are amenable for the study. Extension of the method to other important protein residues would be useful. Arginine is an ionizable residue often important in protein interaction of anions [3]–[6]. Often in discharge of this role, the structure becomes susceptible to selective modification with α-dicarbonyl reagents, in spite of the expected full protonation of guanidine group due to pKa>12 for protonated Arg, contrasted with pKa ∼7 for protonated His [7]–[9]. The “activation” was proven to involve the protein effects in anion recognition promoting reduction of arginine pKa, to “activate” the residue for modification even at a low pH. Characterization of the chemistry and kinetic susceptibility of the reaction [10], [11] paved the way for having “reactive” arginine harnessed as a kinetic reporter of protein microenvironment in functionally critical centers. In this study we apply the method to 2-hydroxyacid dehydrogenases and thus assess possible basis of their specialization as NAD^+^-producing (oxidative) or NADH-producing (reductive) enzymes. The specialization, being critical for metabolism and homeostasis across organisms, evokes interest in elucidation of its structural basis in the enzyme proteins.

2-Hydroxyacid dehydrogenases are NAD^+^/NADH dependent enzymes of wide distribution among prokaryotes and eukaryotes [12]. The family encompasses the enzymes that while diverse in structures and evolutionary histories are identical in their catalytic mechanism and yet distinct in their specificity as oxidative or reductive enzymes. Lactate dehydrogenases (LDHs) and malate dehydrogenases (MDHs) are the prototypical 2-hydroxyacid dehydrogenases relatively better characterized in their redox variations. LDH is a tetramer over two kinds of polypeptide chains, M and H, which are random in their association as tetramers [13]–[15]. The M4 and H4 variants, the extremes of the association, are recognized for their role in *Cori* cycle involving flow of energy charge at organism level on basis of pyruvate/lactate coupled inter-conversion between NAD^+^ and NADH [8]. MDHs are homodimers that occur as cytosolic and mitochondrial variants. The variants are reciprocal in specificity as oxidants/reductants of NAD^+^/NADH with malate/oxaloacetate as the substrate couple. The functional reciprocity of the isozymes is critical for flow of energy charge in eukaryote cells across mitochondrial membrane [16], [17]. Evidently, 2-hyroxyacid/2-ketoacid couple is crucial to metabolism involving nicotinamide coenzymes across biological divisions [8]. In eukaryotes, the enzymes apparently are adapted for aerobic or anaerobic mode of metabolism; prokaryotic enzymes may be similarly adapted for aerobes and anaerobes and may involve similar basis of protein structure. 2-Hydroxy dehydrogenases thus evoke interest in elucidation of the basis of their adaption as oxidative and reductive variants.

Involvement of NAD^+^/NADH as the coenzyme couple raises the possibility that 2-hydroxyacid dehydrogenases may involve electrostatics of cationic or neutral nicotinamide as basis for specialization as redox enzymes. It is possible that the protein effects involved in promotion of preference for oxidized or reduced coenzyme collaterally also “activate” arginines. 2-Hydroxyacid dehydrogenases feature several arginines in ligand binding and enzyme catalysis functions [18], [19]. The enzymes are susceptible to α-dicarbonyl reagents and are inactivated with modification of apparently a specific active site arginine. On evidence of protection against inactivation, LDH appears to have the modified arginine involved in substrate binding [10] by interaction with their common carboxyl function. This would place the residue in close proximity of nicotinamide-ring structure being donor/acceptor of hydride to and from the carbon in substrates adjoining carboxyl function. Thus the effects mediating protein preference for cationic (oxidized) or neutral (reduced) coenzyme may be involved in arginine “activation”; conversely, “activated” arginines may allow the physical basis of redox specificity of the enzymes to be probed kinetically. Reactive arginine as a phenomenon is important, however in this paper it is used as a tool to document and experimentally extract the electrostatics behind coenzyme preferences of 2-hydroxyacid dehydrogenase. Nevertheless clear demonstration of a uniquely modifiable arginine using radioactive PGO in this study sensitive to stoichiometric inactivation of enzymes provides credence to the notion of link between arginine reactivity and redox specificity. Accordingly, 2-hydroxyacid dehydrogenases of prokaryote and eukaryote origin are tested in their “reactive” arginine in possible relevance for redox specificity of the enzymes. The results affirm that the effects activating arginines are varied between specific enzymes and may be critical in specificity of the structures as reductive and oxidative variants.

## Materials and Methods

### Materials

Porcine and rabbit H4 and M4 LDHs, porcine cytosolic and mitochondrial MDHs, *Thermus flavus* MDH, and D-LDHs from *Leuconostoc mesenteroides*, *Staphylococcus epidermidis*, and *Rhizopus oryzae* were purchased from Sigma Chemicals. Sephadex G-50 was from Pharmacia. *Lactobacillus casei* (strain ATCC 7469) was obtained from BARC, Mumbai. All other chemicals were of analytical grade.

### Spectrometric and pH measurements

All chemical modifications and enzyme inactivations were performed at 27°C in the dark. Spectrometric measurements were at 27±0.1°C. pH measurements were with a combination electrode pre-calibrated at pH 4.0, 7.0, and 9.2.

### Buffers

pH and salt titration experiments were in HEPES (pH 6.5 to 8.0) and Bicine (pH 8.5 to 9.5) buffers (50 mM). Adjusting pH initially with 1N NaOH or HCl, and estimating ionic compositions from pKas, ionic strengths were adjusted to 0.04, 0.09, 0.16, 0.25, 0.49, 0.70, and 1.0 with NaCl. Finer pH readjustments, when needed, were done with 1N NaOH or HCl. Possible eventual variations of ionic strength were always within ±3%.

### LDH isolation

Grown as reported [20], *L. casei* cells were harvested after 18 hr, sonicated, centrifuged, and submitted to i) ammonium sulphate fractionation, ii) Sephadex-Blue-F3GA chromatography, and iii) Sepharose-Oxamate chromatography, for which the affinity matrices were prepared as reported [21], [22]. The LDH-enriched ammonium-sulfate fraction was applied in phosphate buffer (50 mM, pH 7.0) to Sephadex-Blue column pre-equilibrated with phosphate buffer (20 mM, pH 7.0) at 4°C. After washing with five column volumes, the matrix was eluted with NADH (0.2 mM) and NaCl (0.3 M). LDH was recovered from the column exactly after one column volume. This partly enriched eluate was applied to Sepharose-Oxamate column pre-equilibrated with phosphate buffer 0.5 M in NaCl and 0.2 mM in NADH. On washing with equilibration buffer, and on elution by omitting NADH, LDH was recovered exactly after one-column volume. Polyacrylamide gel electrophoresis with and without SDS was used for test of purity of the enzyme protein.

### BGn modification

Butylguanidine (BGn) is a reference molecule that was used to measure the arginine pKa changes and the protein microenvironment effects involved in changing pKa's. The BGn (10–100 mM) and phenyl glyoxal (PGO) (0.1 mM) were reacted in suitable buffers and reaction kinetics was monitored with disappearance of absorbance at 254 nm due to PGO. The reactions were allowed to proceed to completion and on this basis correction was made in every absorbance value for contribution of the accumulated product.

### Enzyme inactivation

Prior to inactivation, enzymes were desalted over Sephadex G25 pre-equilibrated in HEPES or Bicine buffer of requisite pH and ionic strength. Enzymes were adjusted to the requisite final dilution, 0.5 or 1.0 unit in the final assay volume. PGO was added from a concentrated stock to 10 mM concentration against LDHs and 5 mM concentration against MDHs. Aliquots drawn periodically were assayed for residual enzyme activity. Parallel controls lacking in PGO were run to assess and correct for, if required, instability of the enzyme in extraction of kinetics of inactivation due to arginine modification under specified condition of pH and ionic strength.

### Enzyme assays

All assays were performed at 27°C in 3 ml final volumes, and were initiated with addition of 50 µl aliquots of enzyme alone or enzyme+inhibitor. The resultant dilution of PGO terminated enzyme inactivation. Enzymes were monitored at 340 nm based on NAD(H) oxidation or NAD^+^ reduction. LDH assays were in 5 mM potassium phosphate buffer (pH 7.5) which was 0.2 mM in NAD(H) and 2 mM in sodium pyruvate; MDH assays were in 0.12 M glycine buffer (pH 10.0) which was 6.3 mM in L-malate and 2.7 mM in NAD^+^.

### Measurement of stoichiometry of modification

M4-LDH (0.2 mg/ml), H4-LDH (0.1 mg/ml), cytoplasmic-MDH (0.4 mg/ml), and mitochondrial-MDH (0.15 mg/ml) were incubated with 10 mM ^14^C-PGO (adjusted by assuming ε = 11,600 mol^−1^ cm^−1^ at 249 nm) in HEPES buffer (50 mM) at pH 8.0. Radioactivity incorporated into protein was measured, as described below, in one 20 µl aliquot, and residual enzyme activity was measured in another 20 µl aliquot, sampled in parallel, as described above. For measurement of radioactive incorporation, aliquots were blotted on a 3 mm Whatmann filter paper disc. The disc was then soaked in chilled 10% TCA (30 min), then 5% TCA (15 min), rinsed with 5% TCA, then 95% ethanol, oven dried, and counted in 0.4% PPO and 0.01% POPOP in 5 ml Toulene. Protein concentration measurements were with the Bradford method.

### Analysis of protein-structure distribution of the charged residues

Sequence alignments were performed with FASTA [23]. Structure-coordinates of porcine H4 and M4 LDHs and of cytoplasmic and mitochondrial MDHs were retrieved from PDB. The coordinates of Cα atoms in correspondence of cationic and anionic residues (Arg, Lys, His, Glu and Asp) were evaluated in their radial distances from coordinates of Cα atom of R171 in LDH, R152 in cytoplasmic MDH, and R161 in mitochondrial MDH [19], [24]. The residues were binned into 5 Å blocks of distance from the reference arginine in each protein. The frequencies of cationic and anionic residues were compared for assessment of isozyme specificity of the proteins.

### Calculation of electrostatics

The structural coordinates 4MDH, 1MLD, 9LDT, 5LDT, and 1LLC downloaded from PDB [25] and stripped of non-protein atoms including water were submitted to finite difference Poisson-Boltzmann calculation using Delphi [26], [27]. Charges were assigned from GROMOS [28], [29]. Charges on ionizable groups were assigned in correspondence to that expected at pH 7.0 for intrinsic pKas of specific residues. Thus arginine (for intrinsic pKa = 12.0) has +0.5 on Nη1 and Nη2, lysine (pKa 10.1) has +1.0 on Nξ, histidine (pKa 6.5) has +0.5 on Nδ1 and Nε2, aspartic acid (pKa 4.5) has −0.5 on Oδ1 and Oδ2, and glutamic acid (pKa 4.5) has −0.5 on Oε1 and Oε2. Dielectric constants 80 and 4 were assigned, respectively, for solvent and protein. The electrostatic potential operative at centroid of guanidinium groups of particular arginine was calculated.

### Calculation of ΔpKa

The calculated electrostatic potentials (mV) at specific GnH^+^ groups were converted into ΔpKas using the formula [30] ΔpKa = e Δφí/2.303k_B_T; φí as electrostatic potential, k_B_ as Boltzmann constant and T as temperature (298 K).

## Results

### Reactive arginine

**Figure 1:**
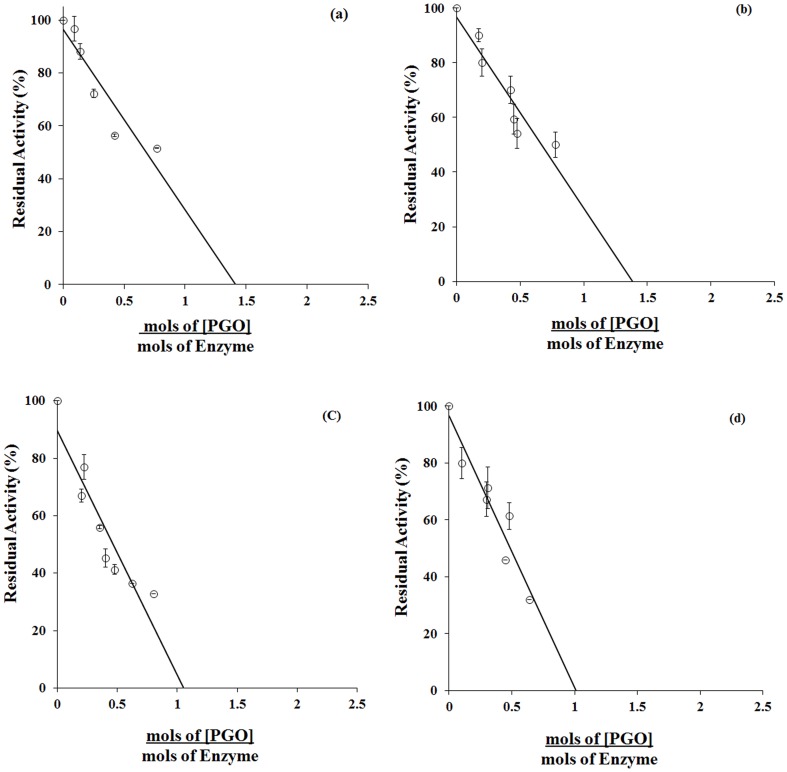
Stoichoimetry of arginine modification and enzyme inactivation. Porcine M4 LDH (**Panel a**), porcine H4 LDH (**Panel b**), mitochondrial MDH (**Panel c**), and cytoplasmic MDH (**Panel d**). Enzymes incubated with ^14^C-PGO were evaluated for radioactivity incorporated and enzyme activity remaining as a function of time.

All LDHs and MDHs are susceptible to inactivation with PGO. In Figure 1 Panels a–d, a 1∶1 correspondence is noted between moles of radio-labeled PGO incorporated and extent of enzyme inactivated, observed that result in panel (a) and (b) enzyme activation do not reach 60%. Clearly, modification of a single arginine promotes stoichiometric inactivation of the enzymes. The initial rate of enzyme inactivation presumably measures reactivity of a functionally critical arginine. Identity of the arginine was probed by assessing effect of pre-equilibrating enzymes with specific ligands. Partial protection from inactivation was evidenced in each enzyme with a specific substrate or coenzyme ligand. The results (not shown) are conformed to published reports [19], [31], [32] according to which modification of an active site arginine inactivates the enzymes. R171 the residue anchoring substrate carboxyl according to crystallographic evidence [33] could be the modifiable arginine. While the arginine in MDHs has not been rigorously identified, it could be the residue congruous with R171 of LDH possibly similar in the basis of “activation”. According to crystallographic evidence [31], R161 in cytosolic MDH and R152 in mitochondrial MDH is the residue anchoring common carboxyl function of substrates in the enzymes. The arginines evoke interest in relevance of their reactivity for redox specificity of 2-hydroxyacid dehydrogenases.

### Reactivity of Arginine

Enzymes distinct in redox specificity may be distinct in “reactivity” of a specific arginine. We assess “reactivity” with butylguanidine (BGn) as the ruler. The structure models arginine side chain and may be applied for assessing not only “reactivity” of arginine but also the activating effects of protein structure. We assess fold activations, viz., *F* values, under specific conditions of buffer, pH, and ionic strength, relative to the kinetic ruler. The *F* value represents the ratio of k_2app_ in enzyme inactivation to the k_2app_ in BGn modification. The observed modification rates (K_obs_) of BGn are pseudo first order in PGO concentration and zero order in BGn concentration, while the observed enzyme inactivation rates (K_obs_) are pseudo first order in enzyme concentration and zero order in PGO concentration. For assessment of *F* values we derived apparent second order rate constants (k_2_app, in units of Mol^−1^ min^1^) as k_obs_/[BGn] in BGn modification and k_obs_/[PGO] in enzyme inactivation. k_2_app values in enzyme inactivation are ranged between 0.66–112 Mol^−1^ min^1^ while in BGn modification they are ranged between 0.47–13.3 Mol^−1^ min^1^ depending on pH and ionic strength. The *F* values derived under specific pH and ionic strength conditions are listed in the Table S1. In prokaryotic LDHs contrasts are noted in the magnitude of *F* values. *L. mesenteroides* and *R. oryzae* LDH has comparatively larger *F* values compared to *L. casei* and *S. epidermidis* LDH indicating a larger sensitivity to pH and ionic strength. The *F* value of *R. oryzae* at pH 7.0 and pH 8.0 are the largest among all the prokaryotes indicative of much larger arginine activation. The *F* values of the muscle form in LDH, mitochondrial MDH and aerobic prokaryotes shows a larger arginine activation as compared to their corresponding isozymes (heart LDH, cytosolic MDH and anaerobic prokaryotes). The variability of arginine activation in isozymes may be due to the difference in the active site electrostatic fields in M4 and H4 LDH and in cytoplasmic and mitrochondrial MDH.

### ΔpKa in “reactive” arginines

**Figure 2:**
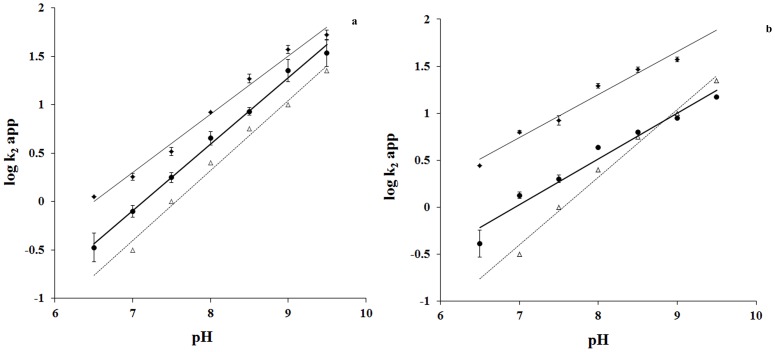
pH dependence of enzyme inactivation and butylguanidine (BGn) reaction rates. Porcine (**Panel a**) and Rabbit (**Panel b**) M4 LDH (♦) and H4 LDH (•), BGn (Δ). Enzyme (1 unit) and PGO (5 mM), BGn (10–100 mM) and PGO (100 µM), were reacted in HEPES (pH 6.5 to 8.0) and Bicine (pH 8.5 to 9.5), buffers (50 mM, Ionic strength 0.05) and reactions were monitored as described under the **Materials and Methods** section. Log k_2_app derived as k_obs_/[PGO] and k_obs_/[BGn] are plotted against pH. Each point is a mean of three independent determinations ± standard error (best fitted data is plotted for BGn, reference molecule). **Panel a** of this figure is reproduced from reference 10 of this article under a CC BY license, with permission from ACS, original copyright 1998.

Arginine activation has been attributed to reduction of pKa in a cationic intermediate on the pathway of modification reaction. We estimate ΔpKa in activation of specific arginines relative to BGn. Results of relevant experiments are summarized in Figure 2 Panels a and b. Log k_2_app vs. pH plots are linear for the reaction of PGO with both BGn and enzymes. The plots in enzyme inactivation are varied in slope, which ranges from 0.25 to 0.70 as is noted in the Table S2. The diminished slopes, relative to the model, suggest that protein pKas could be strong or weak in dependence on acid-base groups in titration of their charge with pH. Independent of slopes, log k_2_app vs. pH plots are always shifted to a lower pH for arginines than for BGn: arginines are effectively diminished in pKa relative to BGn. We calculate ΔpKa's to common reference pH graphically by assessing the requirement of pH change to “activate” BGn to the level of reactivity in specific arginines at pH 7.0. ΔpKa's thus calculated for specific enzymes are listed in the Table S2. Among eukaryotic enzymes, pKa's are more strongly diminished for M4 LDHs and mitochondrial MDH than for H4 LDHs and cytoplasmic MDH. Among prokaryote enzymes, pKa's are more strongly diminished for LDHs of anaerobes *L. mesenteroides* and *S. epidermidis* than for aerobe *L. casei*. ΔpKa is exceptionally large for aerobic *R oryzae* LDH. Considering that the effects in redox specificity of the enzymes may be critical, we address the effect in their likely basis of protein structure.

### Protein effects activating arginines

**Figure 3:**
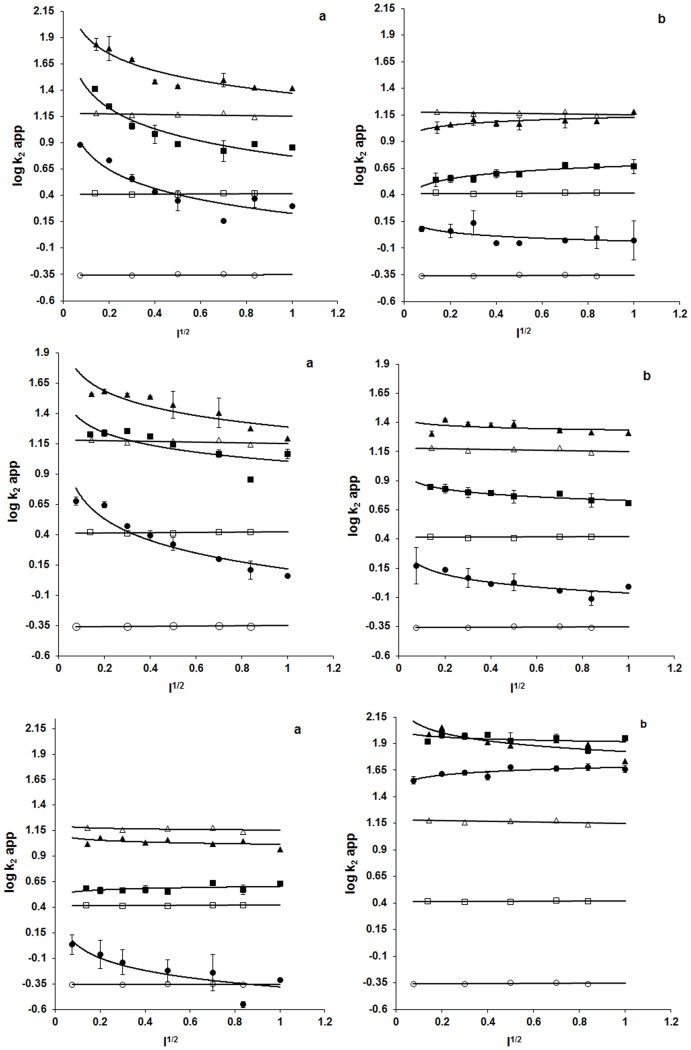
Ionic strength dependence of kinetics of inactivation. Rabbit M4 LDH (**upper panel a**) and Rabbit H4 LDH (**upper panel b**), *L. mesenteroides* LDH (**middle panel a**) and *S. epidermidis* LDH (**middle panel b**), *L. casei* LDH (**lower panel a**) and *R. oryzae* LDH (**lower panel b**) (filled symbol), and kinetics of BGn modification (open symbol), at pH 7.0 (•), 8.0 (▪) and 9.0 (▴). Each point is a mean of three independent determinations ± standard error (best fitted data is plotted for BGn, reference molecule).

**Figure 4:**
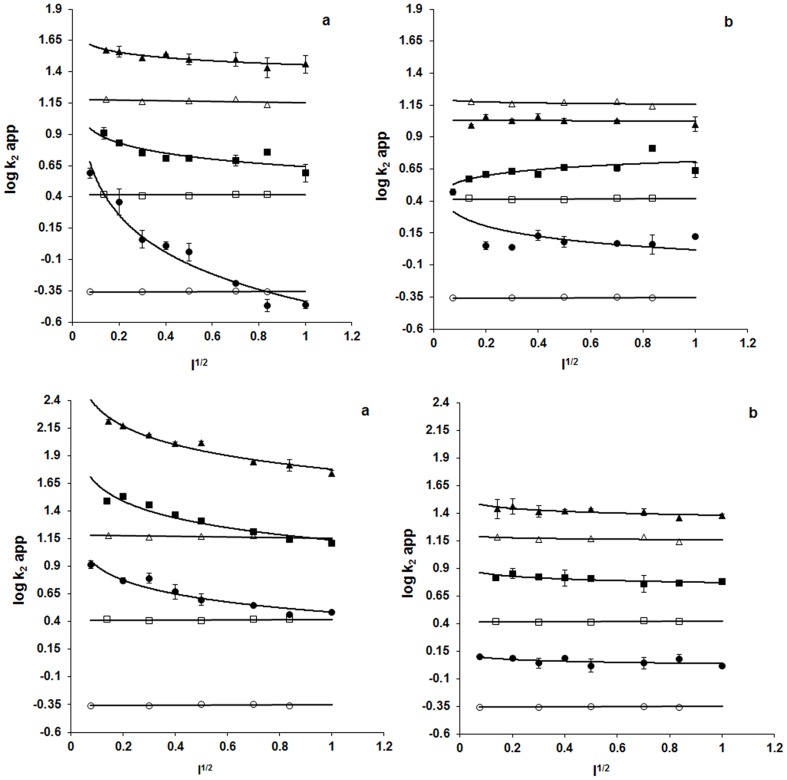
Ionic strength dependence of kinetics of inactivation. Porcine M4 LDH (**upper panel a**) and H4 LDH (**upper panel b**), porcine mitochondrial MDH (**lower panel a**) and cytosolic MDH (**lower panel b**) (filled symbol), and kinetics of BGn modification (open symbol), at pH 7.0 (•), 8.0 (▪) and 9.0 (▴). Each point is a mean of three independent determinations ± standard error (best fitted data is plotted for BGn, reference molecule). The Figure is reproduced from reference 10 of this article under a CC BY license, with permission from ACS, original copyright 1998.

Effects diminishing pKa's are analyzed with ionic strength and pH variation experiments. Moved to protein structure from bulk solvent, arginine side chain can diminish in pKa by destabilizing as guanidinium cation or stabilizing as neutral guanidine with protein effects at the interface with solvent. The effects in principle capable of diminishing arginine pKa, from the level in bulk solvent, are electrostatics and solvation. Electrostatics may be washed out with ionic strength and thus solvation may be unmasked as an activating effect. Arginines are thus analyzed, by titration with ionic strength, in electrostatic activation under specific values of pH. Results in Figures 3 and 4 confirm that having no effect on kinetics of BGn modification at any pH, ionic strength will allow proteins to be examined in electrostatics of arginine activation. At pH 7.0, being our reference pH, M4 LDHs and mitochondrial MDH manifest reduction of arginine reactivity with ionic strength more strongly than H4 LDHs and cytoplasmic MDH. Furthermore, increase of pH from 7 to 8 to 9 diminishes effect of ionic strength in M4 LDHs and mitochondrial MDH. Thus, not only are arginines in M4 LDHs and mitochondrial MDH more reactive than those in H4 LDHs and cytoplasmic MDH, the reactivities have stronger bases in protein electrostatic and the electrostatics may have stronger bases in acid-base groups of the proteins (Figures 3 and 4 Panel a). Contrast of acid-base group participation is evident also in prokaryotic 2-hydroxyacid dehydrogenases (Figure 3 middle and lower panels). Most notably, contribution of ionizable groups is stronger in *S. epidermidis* (anaerobes) and *L. mesenteroides* (anaerobes) LDHs than in *L. casei* (aerobe) LDH. The unusually strong activation of arginine in *R. oryzae* (aerobe) LDH noted in Figure 3 (lower panel) clearly is an example of activation that may have no role for protein electrostatics and thus a basis possibly in solvation environment of the reactive side chain.

### Origins of isozyme-specific electrostatics

**Figure 5:**
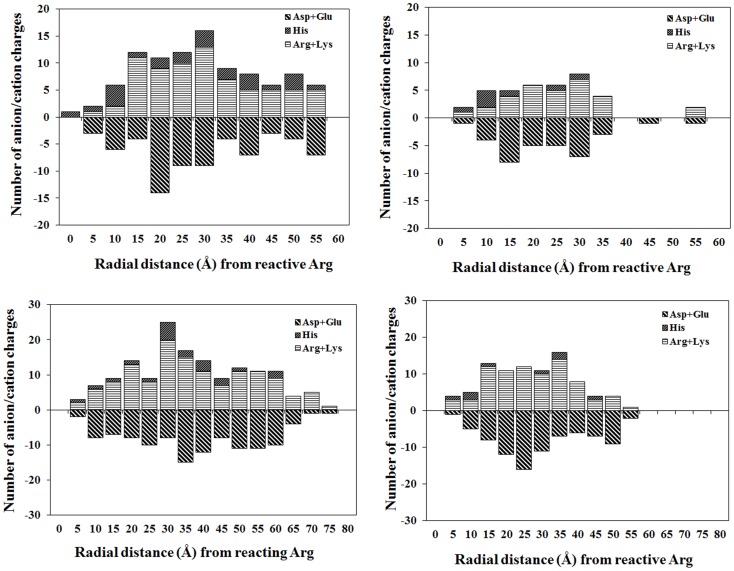
Statistics of ionic residues. Number of cationic (Arg+Lys+His) and anionic (Asp+Glu) residues in specific slabs of distance measured from reactive arginines in the porcine M4 (**upper left**) and H4 (**upper right**) LDH proteins; porcine cytosolic (**lower left**) and mitochondrial (**lower right**) MDH proteins. All distances measured from Cα coordinates of the concerned protein residues.

We examine eukaryote LDHs and MDHs in isozyme specific variations in their acid-base residues. Specifically, analyzing compositional, sequential, and positional details of the residues, we assess consequences for electrostatics of arginine activation. We consider Arg, Lys, and His as the only cations and Asp and Glu as the only anions in analyzing protein charges and their variations with pH. The adaptation of LDHs and MDHs as reductive and oxidative enzymes could involve divergent and convergent evolution: LDH variants have identical folds and possess >75% identical sequences; MDH variants have distinct folds and <20% identity [18], [34]–[36]. Despite possible contrast of evolution, specific isozymes of LDH and MDH share remarkable contrast of composition and placement details of ionizable residues. Compositional data of the residues summarized in the Tables S3 and S4 indicate that the reductive variants in M4 LDHs and cytoplasmic MDHs are higher in proportion of cations than the oxidative variants in H4 LDHs and mitochondrial MDHs. Relevant for electrostatics of reactive arginine will be placement details of cations and anions in folds of the specific isozymes. For analyzing the placements over structurally distinct proteins in reference to the common reactive arginine, we implement a histogram-based approach. We take a distance dependent count of Cα coordinates of cationic and anionic residues considering 5 Å slabs of protein structures starting from Cα of reactive arginine. The slab-wise statistic separately for anions and cations, with the sub-count over His residues highlighted, is presented in Figure 5. While specific isozymes of LDH and MDH are identical or distinct folds, they are isozyme specific in distribution profiles of anion and cation charges. A curious contrast between specific isozymes is that while charges are smeared through the length in M4-LDH and mitochondrial-MDH proteins, the distributions are restricted to initial ∼75% of length in H4-LDH and cytoplasmic-MDH proteins. No conspicuous concentration of charges within specific slabs or conspicuous imbalance of anions or cations is observed in any of the structures. The cations contributing in isozyme specificity of electrostatics in reactive arginine apparently are scrambled over protein structure of the specific isozyme. Even His residues, capable of contributing pH specific variations of charge close to pH of neutrality, are scrambled and do not reflect any preferential enrichment close to reactive arginine.

A sequence-based assessment of cations selective to the reductive isozyme is undertaken with LDH. According to the results summarized in the Table S5, M4 enzyme features several strongly conserved cations that are neutral residues in H4 enzyme. Specific examples include cations in sequence positions 74 (Arg), 134 (Lys), 210 (Lys), 222 (Lys), 266 (Arg), 276 (Lys) and 314 (His/Arg/Lys) in M4 LDH that are neutral residues in H4 LDH. In addition, many sequence positions in M4 LDH are predominantly cations while corresponding position in H4 LDH are predominantly neutral residues.

**Figure 6:**
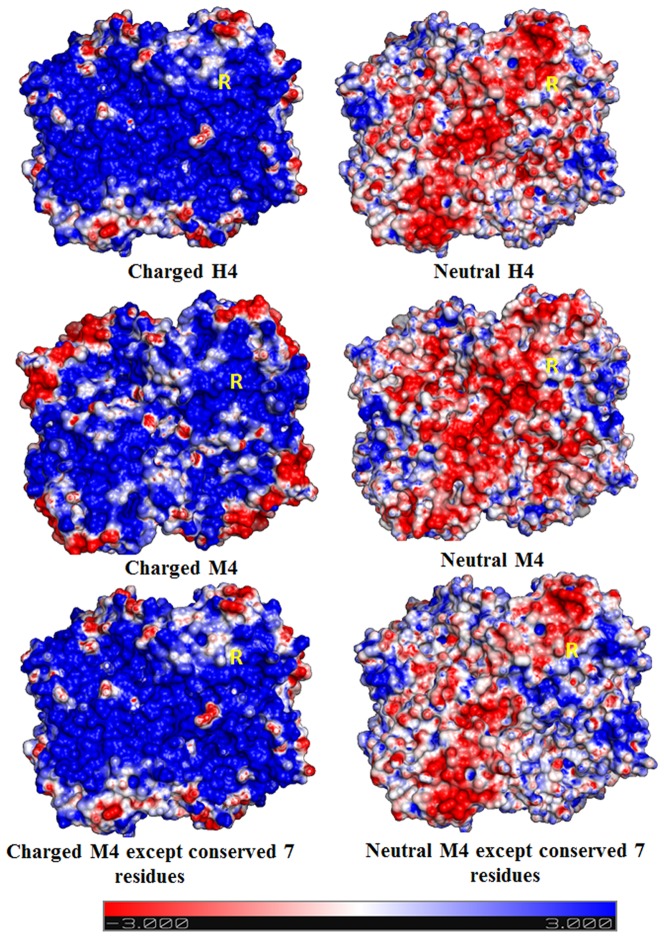
Electrostatic surface potential map. H4 (**upper panel**), M4 (**middle panel**) and conserved amino acid of M4 (**lower panel**), LDH showing effect of protein main chain ionizable side chains (**left**) and effect of only protein main chain (**right**).

We took up calculation of pKa in reactive arginines with finite difference Poisson-Boltzmann calculation using Delphi.[26], [27] The calculations were performed on guanidinium cation of Arg-171 in LDHs and equivalent residues in MDHs. Results in Table 1, indicate reasonable agreement between the pKa changes observed (Table S2) and calculated. The LDH and MDH isozymes being identical or distinct folds were assessed for possibility that electrostatics of arginine reactivity has contributions from peptide dipoles of the main chain folds. The calculations were performed by switching off all side chain charges. Sequence effects involving cation and anion charge were assessed by switching on charges over all cation and anion residues; effects of individual cations specific for the reductive enzyme evaluated with calculation in which the side chain charges were switched on selectively. The results summarized in Table 1 imply that the protein folds are with no role in isozyme specific variation of electrostatics on reactive arginine, and that the variations clearly involve a role for sequences over cation and anion side chains. Results to assess effect of specific cation residues summarized in Figure 6 indicate that conserved cation of M4 isozymes, absent in H4 variant, is dominant in contributing the electrostatics of reactive arginine. Thus the charges defining specificity of reductive isozyme are scrambled over the structures as proteins.

**Table 1:** Calculated ΔpKa's change at reactive arginine of LDHs and MDHs.

Enzyme	Charged	Uncharged	Neutral conserved cation	Charged conserved cation
**LDH**				
Porcine (M4)	−0.75	−0.81	−0.83	−0.73
Porcine (H4)	−0.69	0.51		
*L. casei (aerobic)*	−0.47			
**MDH**				
Porcine (Cyto)	−0.78			
Porcine (Mito)	−1.3			

## Discussion

In post genomics research there are new avenues for addressing long standing problems. Protein structure adaptation for catalysis of specific metabolic processes is an important and unfinished research problem. The problem benefits by the integrative approach of combining inputs of experiment and theory with those of structure and sequence analysis. NAD^+^/NADH redox couple is apparently critical to anaerobic and aerobic metabolism across the biological division of eukaryotes and prokaryotes. The specific roles of the couple presumably are determined by adaptation of 2-hydroxyacid dehydrogenases as oxidation and reduction specific enzymes. Prokaryotic 2-hydroxyacid dehydrogenases have not been specifically classified but may similarly be oxidation and reduction specific due to similar effect of their protein structures. The adaptation remains in basis of structure and underlying physics an unsolved problem. Considering that the reduced and oxidized form of coenzyme is distinct in electrostatics of nicotinamide-ring structure, we have been assessing the distinction in its likely role in the basis of 2-hyroxyacid dehydrogenases as reduction and oxidation specific variants. Recently we reported analysis of protein structural consensus in binding pocket of the cationic form of nicotinamide-ring structure; the consensus cross a distance-based cutoff was proven to illuminate the interaction specificity of oxidized nicotinamide in its likely basis [37]. In the present study we approached the problem with arginine as a kinetic reporter of protein electrostatics.

2-Hydroxyacid dehydrogenases catalyze flow of energy charge in form of NAD^+^/NADH couple over identical catalysis mechanism but distinctive specificity as oxidative and reductive variants [38], [39]. In human M4-LDH has overall positive charge at physiological pH (pI, 8.4) whereas H4-LDH shows an overall negative charge (pI, 5.7). This overall charge differential can further explain the different relative affinities that these two isoforms have for NADH/NAD^+^ and, therefore, their predominant reductive/oxidative nature [40]. Other study on human lactate dehydrogenase isoforms revealed that tetrameric structure of human LDH was appropriate model [41] and two isoforms differs by 0.94 pH unit in pKa between M4 and H4 isozymes and this difference might have arised due to peripheral charged surface residues responsible for distinct activity of these enzymes [42]. Binding isotope effects and ligand and cofactors binding affinities (NADH/NAD^+^, oxamate, pyruvate, L-lactate, and D-lactate) have shown that human M4 and H4 isozymes have distinct active site pockets, H4 isozyme has best binding affinity for NADH cofactor and tightly bind with pyruvate compare to M4 [43], [44]. Catalytic similarity in structurally distinct proteins was underscored when LDH was converted into MDH with only three sequence mutations of the structure [45]. While identical in mechanism the isozymes of both LDH and MDH are distinct in their ligation preferences; M4 LDH and cytosolic MDH are in internal equilibrium constant 4 fold favorable for NADH-pyruvate binding, while H4 LDH are 3 fold favorable in NAD^+^-lactate binding [8]. The enzymes feature an obligatory arginine as an anchor for α-carboxyl function of the common 2-hydroxyacid substrates [30], [32]. The analogous residue is R171 in LDHs, R152 in mitochondrial MDH, and R161 in cytoplasmic MDH. R171→K mutation in LDH was reported to change in binding energy for pyruvate by +5.5 kcal M^−1^ [46]. The arginine is in close proximity of the protein site of nicotinamide-ring binding and is susceptible selective modification with an α-dicarbonyl reagent [10], [47]. Exploiting the residue as a kinetic probe we assessed if redox specific of enzymes may depend on electrostatics of the protein structures.

We found results of experiment, computation, and structure and sequence analysis of diverse 2-hyroxyacid dehydrogenases conformed to the notion that electrostatics could be critical to determination of redox specificity of the enzymes. We found the enzymes inactive stoichiometrically with modification of specific active site arginine. We found the arginine activated to varied extend between specific isozymes of eukaryote structure and similarly varied in activation in prokaryote structures. Analysis of activation with ionic strength and pH titration experiments proved that contrasted activation of arginine was an effect of protein structure which was specific for the oxidative and reductive isozymes in LDH and MDH structures. The possibility that the discrimination between NAD^+^ and NADH could be electrostatics in the basis was confirmed by analysis of charges of protein structures and their distribution specificity in the reductive and oxidative enzymes. Electrostatic calculations with DelPhi [26], [27] affirmed indeed that arginines contrasted involvement of electrostatic in their activation. We found that the contrasts do not reflect the folds of MDH structure but the compositional and placement details of ionizable residues in the structures of the folded proteins. Based on sequence-homology analysis, several cationic residues possibly contributing in redox specificity of LDH isozymes were identified. Due to the limited sequence homology, similarly identification of source of isozyme specific variation of electrostatics could not be undertaken for MDHs. However, the protein structures were found to share a remarkably similar distribution profile of charged residues with respect to position of reactive arginines in the proteins despite the distinct folds of MDH involved. The similar distribution of charges over distinct folds of protein structures has implied that the placements in sequences may be guided by the consideration of electrostatics of the folded structure.

## Supporting Information

Table S1 **Arginine reactivity against BGn as the kinetic ruler.** *F* values [*k*_2app_ in enzyme inactivation/*k*_2app_ in BGn modification] at specific pH 7.0, 8.0, and 9.0.
(DOCX)

Table S2 **Slopes and ΔpKa's calculated from pH dependence of reaction rates.**
(DOCX)

Table S3 **Statistics of ionic residues.** Number of cationic (Arg+Lys+His) and anionic (Asp+Glu) residues and net charge because of the residues on protein subunit of specific LDH.
(DOCX)

Table S4 **Statistics of ionic residues.** Number of cationic (Arg+Lys+His) and anionic (Asp+Glu) residues and net charge because of the residues on protein subunit of specific MDH.
(DOCX)

Table S5 **Residues in specific sequence positions of LDHs.** Positions strictly conserved in a residue are underlined, and those conserved only in cationic charge are italicized.
(DOCX)
