# Correction: Redox Specificity of 2-Hydroxyacid-Coupled NAD^+^/NADH Dehydrogenases: A Study Exploiting "Reactive" Arginine as a Reporter of Protein Electrostatics

**DOI:** 10.1371/journal.pone.0154163

**Published:** 2016-04-18

**Authors:** Pooja Gupta, Mohamad Aman Jairajpuri, Susheel Durani

There is an error in affiliation 1 for authors Pooja Gupta and Susheel Durani. Affiliation 1 should be: Department of Chemistry, Indian Institute of Technology Bombay, Mumbai, India.
