# Supplementary material for: Redox Specificity of 2-Hydroxyacid-Coupled NAD+/NADH Dehydrogenases: A Study Exploiting “Reactive” Arginine as a Reporter of Protein Electrostatics
Source: PLoS One. 2013 Dec 31;8(12):e83505. doi: 10.1371/journal.pone.0083505 (PMC3877072; doi:10.1371/journal.pone.0083505)
Supplement: Table S1 — Arginine reactivity against BGn as the kinetic ruler. F values [k 2app in enzyme inactivation/k 2app in BGn modification] at specific pH 7.0, 8.0, and 9.0. (DOCX) [file pone.0083505.s001.docx]

**Table S1:** **Arginine reactivity against BGn as the kinetic ruler.** *F* values [*k*_2app_ in enzyme inactivation/ *k*_2app_ in BGn modification] at specific pH 7.0, 8.0, and 9.0.

| **Enzymes** | **pH 7.0** | | **pH 8.0** | | **pH 9.0** | |
| --- | --- | --- | --- | --- | --- | --- |
|  | **^#^Zero** | **^#^Infinite** | **Zero** | **Infinite** | **Zero** | **Infinite** |
| **LDH** |  |  |  |  |  |  |
| Porcine (M4)* | 22.40 | 0.71 | 4.84 | 1.41 | 3.59 | 2.10 |
| Porcine (H4)* | 6.00 | 2.50 | 0.94 | 2.24 | 0.72 | 0.89 |
| Rabbit (M4) | 32.30 | 3.13 | 18.00 | 1.78 | 11.69 | 1.58 |
| Rabbit (H4) | 2.89 | 1.76 | 0.84 | 1.88 | 0.66 | 1.00 |
| *Rhizopus oryzae* (aerobic) | 64.70 | 112.0 | 37.50 | 28.20 | 9.40 | 1.00 |
| *Lactobacillus casei* (aerobic) | 3.00 | 1.12 | 1.19 | 1.00 | 0.94 | 1.41 |
| *Leuconostoc mesenteroides* (anaerobic) | 24.60 | 2.83 | 14.90 | 3.16 | 7.50 | 6.30 |
| *Staphylococcus epidermidis* (anaerobic) | 3.77 | 1.76 | 3.29 | 1.68 | 1.96 | 1.60 |
| **MDH** |  |  |  |  |  |  |
| Porcine(Cyto)* | 2.85 | 2.31 | 3.14 | 2.14 | 2.77 | 2.00 |
| Porcine (Mito)* | 24.81 | 6.29 | 33.19 | 4.37 | 12.77 | 3.85 |

^#^ The *F* values at zero and infinite ionic strengths, respectively, calculated using *k*2apps corresponding to the intercepts (for zero ionic strength) and the asymptotes (for infinite ionic strength) to the *Y* axis in respective pH profile plots.

* These values are reprinted from reference 10 of this article under a CC BY license, with permission from ACS, original copyright 1998.
