# Supplementary material for: Redox Specificity of 2-Hydroxyacid-Coupled NAD+/NADH Dehydrogenases: A Study Exploiting “Reactive” Arginine as a Reporter of Protein Electrostatics
Source: PLoS One. 2013 Dec 31;8(12):e83505. doi: 10.1371/journal.pone.0083505 (PMC3877072; doi:10.1371/journal.pone.0083505)
Supplement: Table S2 — Slopes and ΔpKa's calculated from pH dependence of reaction rates. (DOCX) [file pone.0083505.s002.docx]

**Table S2:** **Slopes and ΔpKa’s calculated from pH dependence of reaction rates.**

| **Enzyme** | **Slope** | **ΔpKa** |
| --- | --- | --- |
| BGn | 0.71 |  |
| **LDH** | | |
| Porcine (M4)* | 0.60 | -0.98 |
| Porcine (H4)* | 0.57 | -0.68 |
| Rabbit (M4) | 0.46 | -1.59 |
| Rabbit (H4) | 0.49 | -0.59 |
| *Rhizopus oryzae* (aerobic) | 0.25 | -2.80 |
| *Lactobacillus casie* (aerobic) | 0.52 | -0.65 |
| *Leuconostoc mesenteroides* (anaerobic) | 0.52 | -1.38 |
| *Staphylococcus epidermidis* (anaerobic) | 0.56 | -1.10 |
| **MDH** | | |
| Porcine (Cytosolic)* | 0.63 | -0.70 |
| Porcine (Mitochondrial)* | 0.70 | -1.63 |

* These values are reprinted from reference 10 of this article under a CC BY license, with permission from ACS, original copyright 1998.
