# Supplementary material for: Redox Specificity of 2-Hydroxyacid-Coupled NAD+/NADH Dehydrogenases: A Study Exploiting “Reactive” Arginine as a Reporter of Protein Electrostatics
Source: PLoS One. 2013 Dec 31;8(12):e83505. doi: 10.1371/journal.pone.0083505 (PMC3877072; doi:10.1371/journal.pone.0083505)
Supplement: Table S3 — Statistics of ionic residues. Number of cationic (Arg+Lys+His) and anionic (Asp+Glu) residues and net charge because of the residues on protein subunit of specific LDH. (DOCX) [file pone.0083505.s003.docx]

**Table S3: Statistics of ionic residues.** Number of cationic (Arg + Lys + His) and anionic (Asp + Glu) residues and net charge because of the residues on protein subunit of specific LDH.

| **Organism** | **Isozyme** | **Cations** | **Anions** | **Net charge with His as cation** | **Net charge with His as neutral** |
| --- | --- | --- | --- | --- | --- |
| **Eukaryotes** | | | | | |
| Porcine | M4 | 49 | 35 | 14 | 2 |
| Porcine | H4 | 39 | 34 | 4 | -3 |
| Rabbit | M4 | 48 | 35 | 13 | 2 |
| Rabbit | H4 | 29 | 24 | 4 | -1 |
| Human | M4 | 46 | 36 | 10 | 3 |
| Human | H4 | 41 | 40 | 1 | -6 |
| Mouse | M4 | 55 | 34 | 21 | 12 |
| Mouse | H4 | 41 | 40 | 1 | -8 |
| Rat | M4 | 44 | 35 | 9 | 3 |
| Rat | H4 | 41 | 37 | 4 | -3 |
| Chicken | M4 | 57 | 38 | 19 | 1 |
| Chicken | H4 | 42 | 34 | 8 | 0 |
| **Prokaryotes-aerobic** | | | | |  |
| *Streptococcus pneumonia* |  | 18 | 21 | -3 | -6 |
| *Aurantimonas sps.* |  | 53 | 57 | -4 | -15 |
| *Lactobacillus casei* |  | 36 | 40 | -4 | -14 |
| **Prokaryotes-anaerobic** | | | | |  |
| *Lactobacillus brevis* |  | 33 | 38 | -5 | -14 |
| *Lactobacillus helveticus* |  | 42 | 48 | -6 | -14 |
| *Pasteurella trehalosi* |  | 25 | 26 | -1 | -7 |
