# Supplementary material for: Redox Specificity of 2-Hydroxyacid-Coupled NAD+/NADH Dehydrogenases: A Study Exploiting “Reactive” Arginine as a Reporter of Protein Electrostatics
Source: PLoS One. 2013 Dec 31;8(12):e83505. doi: 10.1371/journal.pone.0083505 (PMC3877072; doi:10.1371/journal.pone.0083505)
Supplement: Table S4 — Statistics of ionic residues. Number of cationic (Arg+Lys+His) and anionic (Asp+Glu) residues and net charge because of the residues on protein subunit of specific MDH. (DOCX) [file pone.0083505.s004.docx]

**Table S4: Statistics of ionic residues.** Number of cationic (Arg + Lys + His) and anionic (Asp + Glu) residues and net charge because of the residues on protein subunit of specific MDH.

| **Organism** | **Isozymes** | **Cations** | **Anions** | **Net charge with His as cation** | **Net charge with His as neutral** |
| --- | --- | --- | --- | --- | --- |
| **Eukaryotes** | | | | | |
| Porcine | Mito | 41 | 29 | 12 | 7 |
| Porcine | Cyto | 45 | 46 | -1 | -5 |
| Human | Mito | 41 | 29 | 12 | 7 |
| Human | Cyto | 45 | 41 | 4 | 0 |
| Mouse | Mito | 40 | 29 | 11 | 6 |
| Mouse | Cyto | 45 | 42 | 3 | -2 |
| Bovine | Mito | 41 | 30 | 11 | 6 |
| Bovine | Cyto | 45 | 43 | 2 | -2 |
| Yeast | Mito | 47 | 37 | 10 | 2 |
| Yeast | Cyto | 38 | 34 | 4 | -3 |
| Chicken | Cyto | 46 | 42 | 4 | 0 |
| **Prokaryotes** | | | | | |
| *Escherichia coli*  (aerobic) | Cyto | 31 | 42 | -11 | -13 |
