# Supplementary material for: Redox Specificity of 2-Hydroxyacid-Coupled NAD+/NADH Dehydrogenases: A Study Exploiting “Reactive” Arginine as a Reporter of Protein Electrostatics
Source: PLoS One. 2013 Dec 31;8(12):e83505. doi: 10.1371/journal.pone.0083505 (PMC3877072; doi:10.1371/journal.pone.0083505)
Supplement: Table S5 — Residues in specific sequence positions of LDHs. Positions strictly conserved in a residue are underlined, and those conserved only in cationic charge are italicized. (DOCX) [file pone.0083505.s005.docx]

**Table S5:** **Residues in specific sequence positions of LDHs.** Positions strictly conserved in a residue are underlined, and those conserved only in cationic charge are italicized.

| **Sequence Position** | **M4** | | | | | **H4** | | | |
| --- | --- | --- | --- | --- | --- | --- | --- | --- | --- |
|  | **Por*** | **Rbt*** | **Ckn*** | **Mus*** | **Hmn*** | **Por** | **Rbt** | **Ckn** | **Hmn** |
| 9 | His | His | His | Val | Tyr | Ala | Thr | Ala | Ala |
| 14 | His | His | His | Gln | Gln | Thr | Ser | Ala | Ala |
| 20 | His | Gln | His | Gln | Gln | Asn | Ser | Asn | Asn |
| 74 | *Arg* | *Arg* | *Lys* | *Lys* | *Arg* | Gln | Gln | Gln | Gln |
| 134 | *Lys* | *Lys* | *Lys* | *Lys* | *Lys* | Ile | Val | Ile | Ile |
| ***171*** | ***Arg*** | ***Arg*** | ***Arg*** | ***Arg*** | ***Arg*** | ***Arg*** | ***Arg*** | ***Arg*** | ***Arg*** |
| 210 | *Lys* | *Lys* | *Lys* | *Lys* | *Lys* | Gln | Gln | Gln | Gln |
| 213 | His | His | His | Asn | His | Asn | Asp | Asn | Asn |
| 222 | *Lys* | *Lys* | *Lys* | *Lys* | *Lys* | Ser | Ser | Ser | Ser |
| 224 | His | Gln | Asp | Gln | Gln | Asn | Asn | Asn | Asn |
| 266 | *Arg* | *Arg* | *Arg* | *Arg* | *Arg* | Ser | Tyr | Ser | Ser |
| 276 | *Lys* | *Lys* | *Lys* | *Lys* | *Lys* | Gln | Lys | Lys | Lys |
| 282 | Lys | Lys | Lys | Asn | Lys | Glu | Glu | Glu | Glu |
| 314 | *His* | *His* | *Lys* | *Arg* | *Arg* | Gln | Gln | Gln | Gln |

^*^The abbreviations are: Por = porcine; Rbt = rabbit; Ckn = chicken; Mus = mouse; Hmn = human
